# The Bogus Diploma Traffic

**Published:** 1881-05

**Authors:** 


### ARTICLE VIII.

### *The Bogus Diploma Traffic.*

The confession of Buchanan, the bogus-diploma trader, published in a recent number of the Philadelphia *Record*, is full of items of interest, and offers much for sober reflection. It is well known that a representative of the Philadelphia *Record* was the means of exposing the bogus-diploma traffic in Philadelphia, and eventually, by direct and indisputable testimony, securing the conviction of Buchanan. The story of the pretended suicide of Buchanan, his recapture and sentence, are still fresh in the minds of our readers. When there appears to be no chance of escaping his punishment, and with no other apparent motive than to ease his guilty conscience, he makes confessions of his multifarious wrong-doings. It appears from the statements made by Buchanan that the bogus-diploma business was not only a distinct branch of industry but was a business of immense proportions, having its recognized agents, drummers, go-betweens, and influential advisers. Through this concern alone sixty thousand bogus diplomas have been sold within the last forty years; of these forty thousand were disposed of in Europe. The prices of each of these pieces of parchment varied from ten to two hundred dollars, according to the means and gullibility of the applicant. Nothing was required from the candidate but the money. The representative of the Philadelphia *Record* purchased several of these diplomas without having studied medicine a single day, and without making the slightest pretension to a knowledge of the science. Fac-similes of these documents are published by the gentleman in question, and help to make up an interesting part of the history of a stupendous and barefaced fraud. As Buchanan has nothing to gain one way or the other by his statements, it is fair to presume that they are worthy of some credence. At all events they are corroborated by the documents which he has surrendered to the authorities. As a matter of course many other parties are implicated in the fraudulent dealings. Many a Buchanan graduate who may be quietly plying his calling in this or another State will hardly thank his distinguished "Dean" for dragging him from security by the publication of his name in the list of men who have graduated from one or other of the fraudulent Philadelphia diploma mills. The disclosures in some instances are quite startling, and implicate many parties whose reputation for fraudulent dealing has been heretofore above suspicion. For instance, we find the names of some persons who have been looked upon as respectable eclectic physicians who were the accredited and confidential agents of the notorious "Dean." Altogether the eclectics, as a class, have not added to their reputation by the recent disclosures. The repeated association of their distinctive title with the bogus colleges is, to say the least, unfortunate.

The lessons to be learned from this miserable business are numerous and important. The possibility of the existence of fraudulent diplomas is proven beyond a doubt, as also the extent to which their traffic may be carried on in the heart of a city celebrated for the high character of its universities. The ease with which an act of incorporation for any so called university can be obtained is demonstrated in the history of the foundation of each of the fraudulent concerns. The manner in which legislative investigations can be warded off, the methods of obtaining favorable reports from legislative committees are clearly shown in the confession. The relative value of diplomas is seen to be fixed by arbitrary, ignorant, and bribed legislative action, instead of by a recognized standard of actual medical qualification.

It is true, when public sentiment became thoroughly aroused, the charters of these bogus institutions were annulled; but unless something more is done, there is no guarantee that the history of diploma traffic will not repeat itself. There is some promise, however, that good may come out of these revelations. Already, as an offshoot of the discussion, we hear of the introduction of a medical bill in the Legislature of Pennsylvania, having for its object the fixing of qualifications concerning which there shall be no reasonable question. At the bottom of all is the system of compulsory registration. When this is properly enforced, there is no dodging the issue. Even Buchanan himself acknowledges that when the medical registration law went into force in England, his diploma trade, which had been brisk before in that country, almost ceased.

But there is one thing in obtaining a registration law and another thing in enforcing it. If Pennsylvania follows the example of New York State in the latter respect, those who practice on the authority of bogus diplomas and false licenses can feel quite secure from molestation.—*Med. Record.*

